# Retinal blood flow increases with diabetic retinopathy severity in type 2 diabetes: a cross-sectional study using laser doppler velocimetry

**DOI:** 10.1038/s41598-026-55921-y

**Published:** 2026-06-15

**Authors:** Ami Konno, Akifumi Kushiyama, Mitsuru Otsubo, Shun Konno, Harumasa Yokota, Taiji Nagaoka

**Affiliations:** 1 Department of Ophthalmology, Asahikawa Medical University, 2-1-1-1 Midorigaoka Higashi, Asahikawa, Hokkaido, 078-8510 Japan; 2 Department of Pharmacotherapy, Meiji Pharmaceutical University, Kiyose, Japan

**Keywords:** Diseases, Endocrinology, Medical research

## Abstract

Retinal arterial blood flow is known to be reduced in patients with type 2 diabetes mellitus (T2D) No Diabetic Retinopathy (NDR) compared with healthy individuals, yet the hemodynamic changes across the early stages of diabetic retinopathy (DR) remain unclear. This cross-sectional study investigated the association between retinal blood flow (RBF) and DR severity in patients with T2D using laser Doppler velocimetry, which provides absolute measurements of retinal arterial diameter, flow velocity, and volumetric blood flow. A total of 419 participants were included and classified as NDR (n = 323), mild non-proliferative DR (NPDR; n = 60), moderate NPDR (n = 8), or severe NPDR (n = 28). Median RBF increased progressively with DR severity, measuring 9.8, 10.6, 11.9, and 11.6 μl/min, respectively. Trend analysis using the Jonckheere–Terpstra test demonstrated a significant positive association between RBF and DR stage (p = 0.035). These findings suggest that while RBF is reduced in the earliest stage of diabetes, it subsequently increases with worsening retinopathy, potentially reflecting impaired retinal autoregulation and pressure-dependent hyperperfusion. Measurement of retinal arterial blood flow may therefore serve as a potential hemodynamic marker associated with DR severity in T2D.

**Supplementary Information:**

The online version contains supplementary material available at https://doi.org/10.1038/s41598-026-55921-y.

## Introduction

Diabetic retinopathy (DR) is a leading cause of vision loss in the working-age population globally. Vascular dysfunction plays a crucial role in its pathogenesis, and changes in retinal blood flow (RBF) have been reported in patients with diabetes mellitus (DM)^1^.^2^Several noninvasive imaging techniques have been developed to evaluate ocular circulation, including optical coherence tomography angiography (OCTA) and laser speckle flowgraphy. However, these modalities primarily provide qualitative or relative blood flow measurements. In contrast, laser Doppler velocimetry (LDV) enables quantitative assessment of absolute RBF by simultaneously measuring retinal vessel diameter and blood flow velocity^3^. Previous clinical studies using LDV have revealed that patients with type 2 DM (T2D) without clinically evident DR (NDR) already demonstrate reduced RBF compared with healthy individuals, indicating subclinical microvascular dysfunction^4^. However, the association between DR severity and RBF remains controversial. Some studies indicate a progressive reduction in flow with advancing DR^5^, whereas others have reported increased blood flow in moderate to severe stages, possibly indicating impaired autoregulation and increased vascular endothelial growth factor (VEGF) and nitric oxide levels^6^, which can increase RBF.

To clarify this complicated association, we conducted a cross-sectional study using LDV to evaluate retinal arterial blood flow in patients with T2D across different DR stages. In this study, we aim to identify whether RBF changes systematically with DR severity and to investigate the effect of systemic factors, including blood pressure, on retinal hemodynamics. Understanding these changes may improve disease progression assessment and help identify patients at higher risk for vision-threatening complications.

## Methods

### Study design and participants

**Fig. 1:**
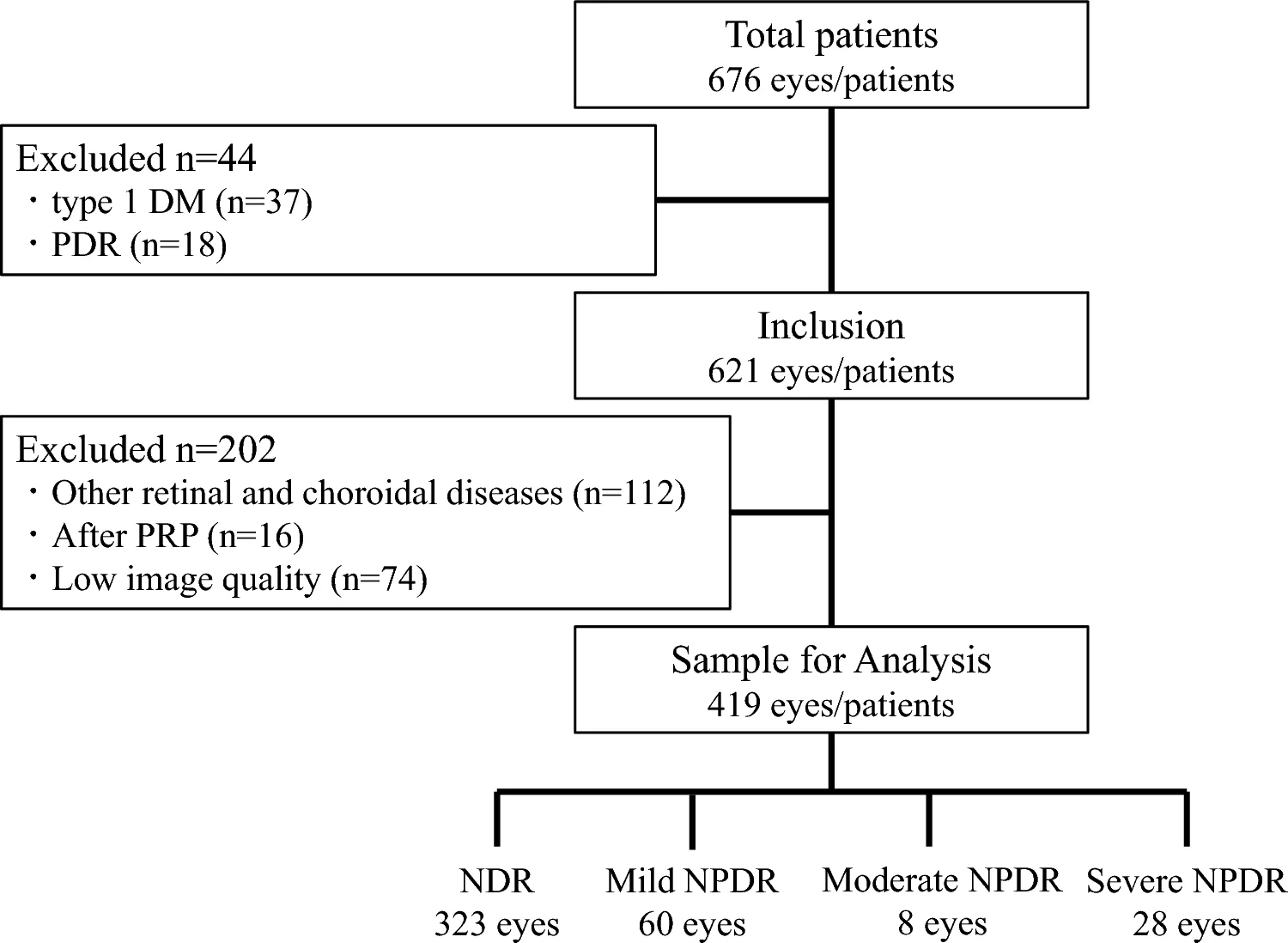
Study population and inclusion process. In this study, 676 eyes from 676 patients were initially screened. Of them, 44 eyes were excluded due to a diagnosis of type 1 diabetes (n = 37) or proliferative diabetic retinopathy (PDR, n = 18), and 202 eyes were further excluded because of other retinal or choroidal diseases (n = 112), previous panretinal photocoagulation (PRP, n = 16), or low-quality OCTA images (n = 74). Consequently, the final analysis included 419 eyes, comprising 323 eyes without diabetic retinopathy (NDR), 60 with mild nonproliferative DR (NPDR), 8 with moderate NPDR, and 28 with severe NPDR.

This cross-sectional study included patients with T2D who visited the Department of Ophthalmology at Asahikawa Medical University Hospital from April 2001 to March 2014. Patient selection was performed in a stepwise manner as follows. First, a total of 676 eyes were screened for eligibility. Among these, eyes with type 1 diabetes mellitus (n = 37) and proliferative DR (PDR) (n = 18) were excluded, leaving 621 eyes that met the initial inclusion criteria, defined as the presence of No Diabetic Retinopathy (NDR) or Nonproliferative DR (NPDR) based on fundus examination. Next, additional exclusion criteria were applied. A total of 202 eyes were excluded due to other retinal or choroidal diseases such as age-related macular degeneration or retinal artery occlusion (n = 112), a history of panretinal photocoagulation (PRP) (n = 16), or insufficient image quality for analysis (n = 74). After applying these criteria, 419 eyes were included in the final analysis (Fig. 1). When both eyes of a patient met the eligibility criteria, the eye with more severe diabetic retinopathy was selected for analysis to avoid inter-eye correlation. The ethics committee at Asahikawa Medical University, Asahikawa, Japan, approved the study protocol that adhered to the Declaration of Helsinki guidelines. Participants comprised native Japanese patients who signed written informed consent before enrollment and after receiving a detailed explanation of the study design and protocol.

### Clinical examinations and systemic parameters

All participants underwent a detailed ophthalmic examination, including best-corrected visual acuity, slit-lamp biomicroscopy, intraocular pressure measurement, and fundus examination using fundus photography. DR severity was classified based on the international clinical DR severity scale^7^.

All participants underwent laboratory assessments. Glycated hemoglobin (HbA1c) was measured employing routine methods. Systolic blood pressure (SBP), diastolic blood pressure (DBP), mean arterial blood pressure (MBP), and heart rate were estimated using an electronic sphygmomanometer (EP-88Si, Colin, Tokyo, Japan). T2D is a chronic metabolic disorder characterized by peripheral insulin resistance and a relative (not absolute) insulin secretory defect. We recruited patients with T2D diagnosed according to American Diabetes Association criteria—defined as individuals receiving insulin or oral hypoglycemic therapy^8^. Further, the duration of T2D was recorded for each patient. Patients receiving antihypertensive drugs were considered to have hypertension.

### RBF measurements

RBF was assessed after completing the ocular examination. To minimize confounding factors, participants refrained from consuming caffeine-containing beverages, including coffee, for at least 12 h before testing. A retinal LDV system (Canon Laser Blood Flowmeter, model CLBF 100; Canon, Tokyo, Japan) was used to evaluate blood flow in the superior branch of the first-order temporal retinal artery, as previously described in detail^9,10^. In summary, this noninvasive method employs a bidirectional LDV technique to identify the absolute velocity of red blood cells (RBCs) along the centerline of the target vessel. The mean retinal artery velocity (V) was defined as the average peak speed of RBCs recorded over a single cardiac cycle. Retinal artery diameter (D) was calculated automatically via computer-based analysis of vessel images captured on the array sensor, with the vessel edges identified at the half-maximal transmittance level. Procedures for fundus observation were standardized across all participants. To ensure measurement accuracy, data were corrected for individual ocular magnification utilizing Littmann’s formula, which incorporates axial length and keratometric values.

### Calculations

RBF was derived using the equation$RBF\, = \,Velocity\, \times \,\left( {Diameter} \right)^{2}$. Here, the velocity was obtained by dividing the averaged maximum velocity by 2. The factor “2” in this calculation comes from Poiseuille’s law^9^, representing assumptions of laminar blood flow. Vessel cross-sectional area was based on arterial diameter measured at the LDV site.

### Statistical analysis

Categorical data were presented as numbers and percentages. The Shapiro–Wilk test was used to assess the normality of quantitative variables, with a *P*-value of greater than 0.05 indicating normal distribution. Variables with normal distribution are presented as mean ± standard deviation, whereas nonparametric data are reported as median and interquartile range. Trend analyses were conducted to evaluate associations between retinopathy severity and these parameters. Simple linear regression was performed to examine the association between MBP and RBF parameters. Multivariable analysis was conducted to identify factors associated with RBF. JMP Pro 17 (SAS Institute Inc., Cary, NC, USA) was used for statistical analyses.

## Results

A total of 419 eyes (61.9%) met the inclusion criteria for quantitative analysis. Among them, 323, 60, 8, and 28 had NDR, mild NPDR, moderate NPDR, and severe NPDR, respectively (Fig. 1). Table 1 summarizes the baseline characteristics of the participants. No significant differences in age or HbA1c were found among the four DR severity groups. However, SBP, DBP, and MBP demonstrated a significant increasing trend with DR severity (*P* = 0.0015, 0.0118, and 0.0025, respectively). The duration of DM was significantly longer in patients with advanced DR (*P* < 0.0001). Regarding medication use, insulin therapy increased significantly with DR severity (*P* < 0.0001). In addition, the use of antihypertensive medications was also higher in patients with more advanced DR (*P* = 0.0025). The prevalence of diabetic nephropathy increased significantly with DR severity (*P* < 0.0001).

**Table 1:** Clinical and physical characteristics in the study groups.

Variables	NDR	Mild NPDR	Moderate NPDR	Severe NPDR	*P* value
Patients, n (men:women)	323 (173:150)	60 (33:27)	8 (5:3)	28 (16:12)	0.6064
Age, years	57.5 ± 11.2	58.7 ± 9.9	53 ± 11.3	54.1 ± 8.4	0.2245
SBP, mm Hg	134.5 ± 20.2	140.6 ± 21.8	145.8 ± 23.2	145.4 ± 21.6	**0.0015**
DBP, mm Hg	74.7 ± 12.6	74.9 ± 12.2	83.4 ± 11.0	80.4 ± 11.9	**0.0118**
MBP, mm Hg	94.6 ± 13.9	96.8 ± 14.3	104.2 ± 14.1	102 ± 14.2	**0.0025**
Duration of diabetes, years	8.0 ± 8.3	11.0 ± 6.0	13.5 ± 6.2	15.0 ± 8.4	** < 0.0001**
HbA1c, %	8.4 ± 2.1	8.0 ± 1.4	8.0 ± 1.0	7.8 ± 2.1	0.2120
Insulin use, n (%)	63 (19.5)	22 (36.7)	2 (25)	9 (32.1)	** < 0.0001**
Antihypertensive medications, (%)	160 (49.5)	40 (75.5)	3 (75)	15 (65.2)	**0.0025**
Diabetic nephropathy, n (%)	9 (2.8)	3 (5.0)	1 (12.5)	13 (46.4)	** < 0.0001**

NDR = no diabetic retinopathy; NPDR = nonproliferative diabetic retinopathy; SBP = systolic blood pressure; DBP = diastolic blood pressure; MBP = mean blood pressure; HbA1c = glycated hemoglobin; HT = hypertension.

All values are presented as mean ± standard deviation. P values for continuous variables were calculated using the Jonckheere–Terpstra test, and those for categorical variables were calculated using the Cochran–Armitage trend test. Bold values indicate statistical significance (P < 0.05).

**Fig. 2:**
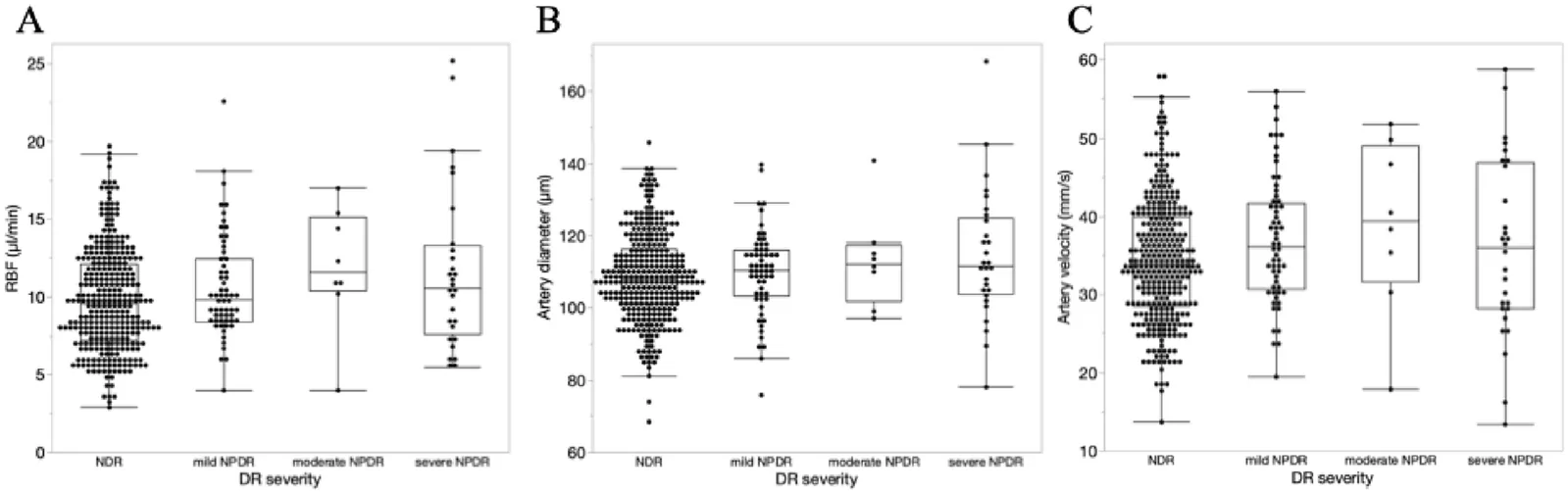
Graphs illustrating the association between diabetic retinopathy severity and retinal blood flow measurements obtained using laser Doppler velocimetry (LDV). Median RBF values were 9.8, 10.6, 11.9, and 11.6 μl/min in the NDR, mild NPDR, moderate NPDR, and severe NPDR groups, respectively. (**A**) Retinal blood flow (RBF) (*P* = 0.0135), (**B**) arterial diameter (*P* = 0.0464), and (**C**) flow velocity (*P* = 0.0312) showed a significant increasing trend with DR severity.

**Fig. 3:**
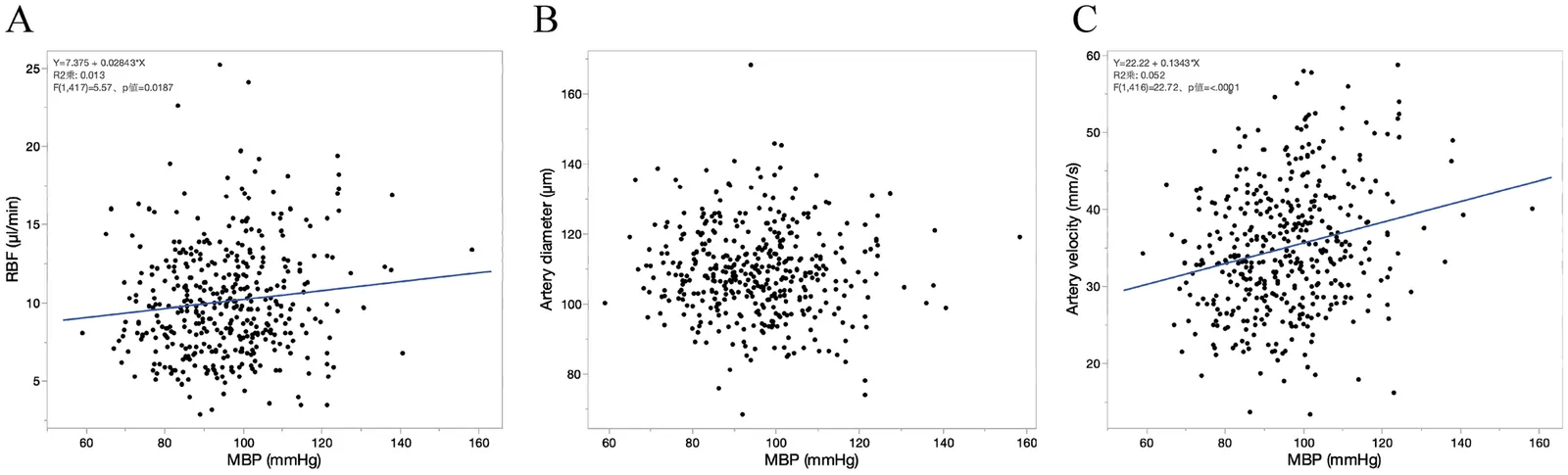
Scatterplots showing the relationship between mean blood pressure (MBP) and the parameters of LDV. (**A**) Scatterplot showing a significant positive correlation between MBP and RBF (β = 0.0284, *P* = 0.0187). (**B**) No significant association was observed between MBP and arterial diameter (*P* = 0.2723). (**C**) Flow velocity was significantly correlated with MBP (β = 0.1343, *P* < 0.0001).

Figure 2 illustrates RBF measurements obtained employing the LDV system. The median retinal arterial RBF values were 9.8, 10.6, 11.9, and 11.6 μl/min in the NDR, mild NPDR, moderate NPDR, and severe NPDR groups, respectively. With increasing DR severity, RBF (*P* = 0.0135), arterial diameter (*P* = 0.0464), and flow velocity (*P* = 0.0312) demonstrated significant upward trends (Fig. 2). Additional analysis combining mild and moderate NPDR into a single group yielded results consistent with the primary analysis (Supplementary Figure S1). Simple linear regression analyses were conducted to evaluate the associations between MBP and RBF parameters (Fig. 3). As MBP increased, both RBF (β = 0.028, *P* = 0.0187; Fig. 3A) and flow velocity (β = 0.134, *P* < 0.0001; Fig. 3C) demonstrated significant positive associations, whereas arterial diameter exhibited no significant change (*P* = 0.2723; Fig. 3B). In summary, increasing DR severity was associated with higher RBF, larger vessel diameter, and faster flow velocity. Further, MBP increased with DR progression.

The multiple linear regression model, including DM duration, MBP, age, sex, and DR severity, explained a modest proportion of variance in RBF. Of all predictors, only DR severity emerged as an independent determinant of RBF (β = 0.615;* P* = 0.0045). MBP demonstrated a positive trend (β = 0.021;* P* = 0.0835), but it did not reach statistical significance. DM duration, age, and sex were not significantly related to RBF (Table 2).

**Table 2:** Multivariable linear regression analysis of factors associated with retinal blood flow.

Predictor variables	Unstandardized β	Lower 95%CI	Upper 95%CI	*P* value
DR severity	0.615	0.192	1.039	**0.0045**
MBP	0.021	−0.003	0.045	0.0835
sex (male = 1)	−0.293	−0.628	0.043	0.0875
Duration of diabetes	−0.003	−0.046	0.040	0.9031
Age	−0.001	−0.033	0.030	0.9285

DR = diabetic retinopathy; MBP = mean blood pressure β represents the unstandardized regression coefficient. Data are presented as β with 95% confidence intervals and corresponding P values. DR severity was treated as an ordinal variable. Bold values indicate statistical significance (P < 0.05).

## Discussion

We revealed that in T2D, RBF, vessel diameter, and flow velocity increase as DR severity progresses. Previous studies in type 1 DM (T1D) have described a biphasic pattern in which RBF initially decreases when disease duration is short, and increases as DR severity and diabetes duration worsen^1^. Similar reports have indicated that RBF increases with worsening DR; however, such studies were limited to patients with T1D^11^and have included small sample sizes^6^. Hommer et al. reported that in patients with T2D, RBF tended to increase with advancing DR severity^12^. However, this trend was not observed in the most severe stages, indicating that further investigation is warranted. PRP reduces RBF^13^; thus, we excluded any eyes that had undergone laser treatment, ensuring that our measurements accurately reflect true retinal perfusion. Furthermore, previous RBF studies have measured venous blood flow, whereas our study derived RBF from arterial diameter and arterial flow velocity. Thus, this work is novel in that it noninvasively assessed arterial blood flow dynamics using LDV in a large cohort of patients with T2D.

Some parameters demonstrated significant changes with increasing DR severity, whereas others did not. SBP, DBP, and MBP all increased as DR progressed. T2D and hypertension frequently coexist; however, increases in both SBP and DBP were correlated with DR severity^14,15^. Furthermore, higher MBP has significantly increased the risk of DR progression^16,17^, and antihypertensive therapy has prevented the onset of DR^18^. In addition, the prevalence of diabetic nephropathy increased significantly with DR severity in the present study. This finding is consistent with previous reports demonstrating a close association between diabetic retinopathy and nephropathy, suggesting that these complications share common microvascular pathophysiological mechanisms^19^. These results support the concept that retinal microvascular alterations may reflect systemic microvascular dysfunction in patients with diabetes. The risk of both DR onset and progression has been confirmed to increase significantly with longer DM duration^20^. In the current study, disease duration was significantly longer in eyes with more severe DR, whereas HbA1c did not differ across DR severity levels. This finding is consistent with previous reports, indicating that HbA1c does not serve as a reliable indicator of DR presence or severity despite its clinical use for DM monitoring^21^. These findings suggest that long-term glycemic exposure has a stronger influence on DR than current HbA1c levels. This supports the concept of metabolic memory, in which the cumulative effects of past hyperglycemia continue to drive retinopathy progression even after glycemic control improves^22^.

RBF, arterial diameter, and arterial velocity increased significantly with DR severity (Fig. 2). Although the number of eyes with moderate NPDR was relatively small, additional analysis combining mild and moderate NPDR yielded consistent results, supporting the robustness of our findings (Supplementary Figure S1). Kohner et al. compared RBF among patients with T1D and T2D and healthy controls^6^. They reported that RBF increases with DR progression; however, their measurements were confined to veins, from which they derived total flow based on venous diameter and flow velocity. They justified this selection by noting that arterial measurements demonstrate large pulsatile fluctuations due to the cardiac cycle, which complicates flow assessment, and that retinal arteries—being end arteries without anastomoses—form a closed‐loop system with their corresponding veins, so arterial and venous flow volumes are effectively equal^23^. In the current study, we focused on arterial measurements because arteries also serve as the primary site of autoregulatory control and provide the most sensitive readout of vasoconstriction and vasodilation in response to perfusion pressure changes. Assessing arteries enables direct quantification of the blood volume delivered to the retina.

In the current study, we revealed that RBF, arterial diameter, and arterial flow velocity all increased with advancing DR severity. In healthy participants, increases in MBP are buffered by intact autoregulation, which keeps ocular blood flow constant^16^. However, DM-induced microvascular damage disrupts this autoregulatory capacity; thus, any increase in perfusion pressure translates directly into pathological hyperperfusion^24^. This hyperperfusion may increase shear stress on the vessel wall, leading to endothelial injury. Consequently, ischemic and exudative changes are exacerbated, thereby driving DR progression. Previous studies using retinal oximetry have demonstrated a reduced arteriovenous oxygen saturation difference in patients with diabetic retinopathy, suggesting impaired oxygen extraction at the tissue level. This phenomenon has been consistently observed across multiple studies. For example, Faryan et al. reported a significant reduction in the arteriovenous oxygen saturation difference in eyes with nonproliferative diabetic retinopathy, indicating impaired oxygen extraction^25^. Our findings of increased retinal blood flow with DR severity may be complementary to these observations. Specifically, increased blood flow in the presence of reduced oxygen utilization may reflect a state of neurovascular and metabolic dysregulation, in which hyperperfusion does not translate into effective oxygen delivery.

Simple linear regression analysis revealed that blood pressure demonstrated a significant positive correlation with both RBF and flow velocity, whereas no association was observed with vessel diameter (Fig. 3). Autoregulatory capacity plays a crucial role in regulating tissue blood flow to ensure a steady delivery of oxygen and nutrients to organs^26^. We previously reported that RBF is autoregulated in response to acute increases in systemic blood pressure^27^. We conducted a cold pressor test to induce a rapid increase in systemic blood pressure and used LDV to evaluate changes in retinal vascular parameters. In response to the acute increase in blood pressure, flow velocity rose along with a transient RBF elevation. However, retinal arteriole vasoconstriction subsequently restored RBF to baseline. These results indicate that arteriolar constriction plays a crucial role in maintaining stable RBF during acute blood pressure elevations^28^. In DM, the coupling between systemic blood pressure and retinal perfusion was further compromised^29^. Epidemiological data from the United Kingdom Prospective Diabetes Study^18^and the Wisconsin Epidemiologic Study of Diabetic Retinopathy^16^, respectively, demonstrate that each 10 mmHg rise in MAP increases the risk of DR progression by approximately 28% and that poorly controlled hypertension accelerates microvascular damage.

Several mechanisms regulate ocular blood flow, including myogenic, metabolic, neurogenic, and endothelium-derived factors^26^. Further, DM has damaged smooth muscle cells, endothelial cells, and pericytes^30^. Thus, the absence of any correlation between blood pressure and arterial diameter in our data indicates impaired arteriolar constriction in response to pressure changes—i.e., autoregulatory function—in patients with DM with advancing DR. Recent reports have shown more severe deep capillary plexus (DCP) perfusion deficits on OCTA and significantly worse visual acuity in eyes with DR complicated by hypertension than in eyes without hypertension^31^. Recent OCTA studies indicate that DCP dropout occurs as the earliest pathological changes in the initial DR stages and progresses more rapidly as the disease advances^32^. Hypertension may disrupt retinal autoregulation, leading to disproportionate perfusion changes in the more vulnerable DCP.

**Fig. 4:**
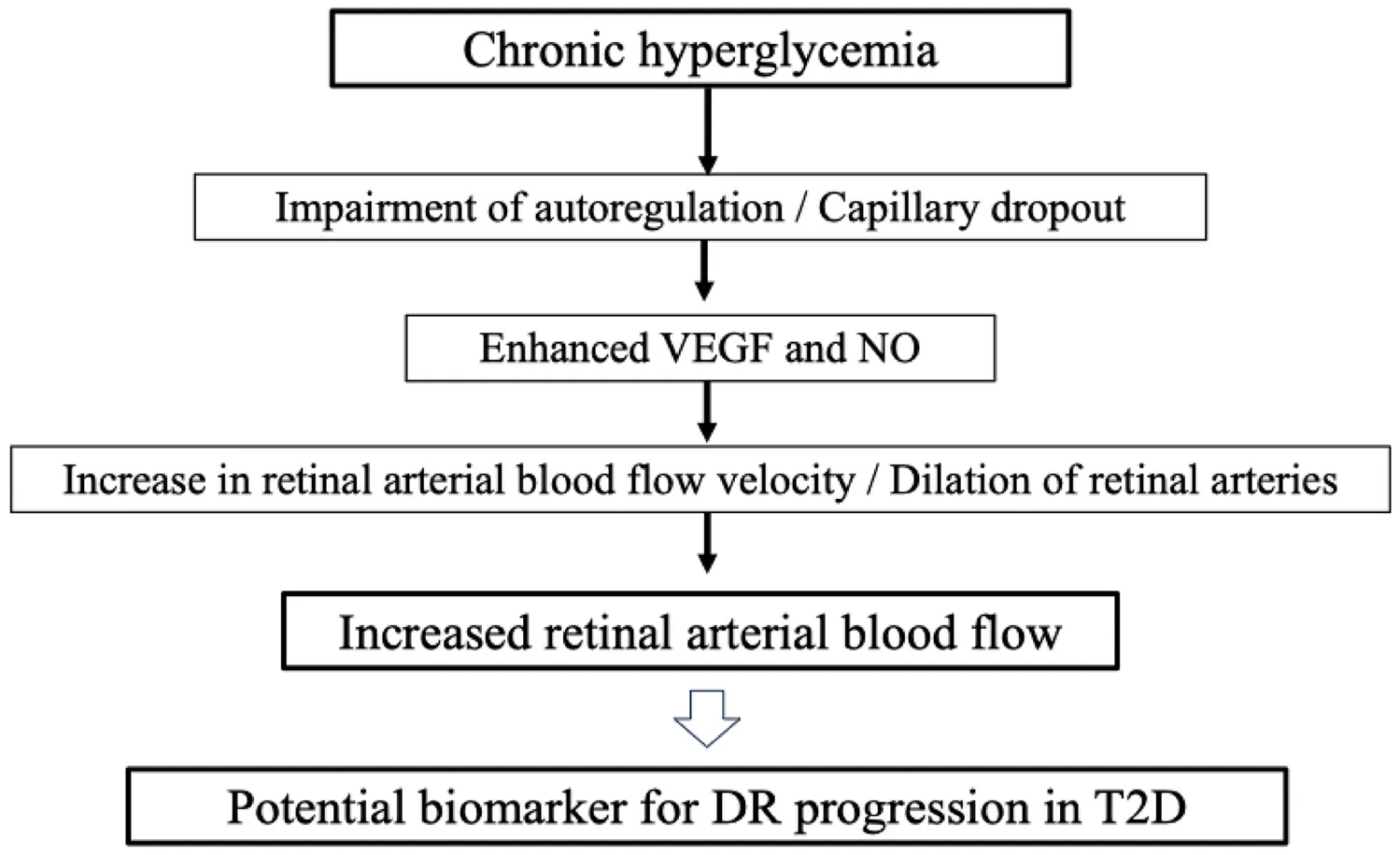
Conceptual framework illustrating the potential relationships among chronic hyperglycemia, increased systemic blood pressure, impaired vascular autoregulation, and altered retinal blood flow in type 2 diabetes mellitus. In this model, the combined influence of hyperglycemia and hypertension may be associated with pressure-driven increases in retinal arterial flow velocity and arterial dilation. These hemodynamic changes may be accompanied by increased shear stress and improved VEGF signaling secondary to capillary dropout, which could facilitate nitric oxide-mediated vasodilation and further changes in retinal blood flow. Collectively, these processes are proposed to provide a mechanistic context in which hyperperfusion of retinal arterioles and ischemic features in the capillaries may coexist during diabetic retinopathy progression.

The hemodynamic pattern of RBF increases with advancing DR severity was striking, with eyes in more advanced DR demonstrating significantly higher RBF than those with no retinopathy, even after accounting for systemic factors. These findings support a long-standing hypothesis by Kohner and Rassam that retinal hyperperfusion due to impaired autoregulation is central to DR pathogenesis^33^. The present results support the concept that impaired autoregulation in T2D enables systemic blood pressure to directly affect retinal hemodynamics. In healthy eyes, transient changes in systemic pressure are compensated by arteriolar constriction, thereby maintaining constant flow. However, in patients with DM, this buffering capacity is attenuated. Rassam et al. revealed that experimentally induced hypertension caused a disproportionate increase in RBF due to defective autoregulation, a mechanism that may accelerate DR progression^24^. Similarly, our recent work and that of others have revealed that MBP is an independent determinant of RBF, indicating that chronic hypertension amplifies retinal hyperperfusion through sustained hemodynamic stress on the microvasculature^4^. However, in the present study, MBP was not identified as an independent predictor of RBF in the multivariable analysis, whereas DR severity remained the strongest determinant. This suggests that although systemic blood pressure contributes to retinal hemodynamics, its effect may be secondary to disease-related microvascular changes associated with DR progression. Ueno et al. further reported that long-standing hypertension promotes structural remodeling and dropout of the DCP, thereby associating systemic pressure exposure with localized retinal ischemia^34^. Taken together, these findings indicate that, in T2D, hypertension may be associated with both early retinal hyperperfusion and subsequent capillary occlusion, according to the conceptual framework illustrated in Fig. 4. Chronic hyperglycemia and elevated systemic blood pressure may act in combination to impair vascular autoregulation, leading to pressure-driven increases in retinal arterial flow velocity and arterial dilation. The accompanying elevation in shear stress^33^, together with improved VEGF signaling secondary to capillary dropout, may facilitate nitric oxide-mediated vasodilation of retinal arterioles^34^, thereby contributing to further increases in RBF. Collectively, these processes may provide a mechanistic context in which hyperperfusion of retinal arterioles and ischemic alterations of retinal capillaries coexist across different DR stages.

This study has some limitations. First, eyes with PDR were excluded because PRP has decreased retinal oxygen demand and reduced RBF^13,35^. Second, as a cross-sectional study, we did not assess long-term hemodynamic changes within individuals. Smoking status was not assessed in this study, and thus its potential influence on retinal autoregulation and blood flow could not be evaluated. One limitation of this study is the potential variability in RBF measurements, which may affect its utility as a standalone biomarker. Importantly, the observed increase in retinal arterial blood flow with advancing DR severity should not be interpreted as a retinal perfusion improvement. Rather, this hemodynamic pattern likely reflects a pathological state characterized by impaired retinal autoregulation, in which RBF becomes increasingly pressure-passive. In such a state, increased arterial flow can coexist with progressive microvascular nonperfusion and tissue hypoxia. Prospective longitudinal investigations are warranted to fully characterize temporal alterations in retinal circulation. However, LDV yields absolute flow values; thus, our comparison of RBF parameters across DR severity in a large cohort remains highly informative. An important limitation of the present study is that the CLBF-100 is no longer commercially available, which may limit the generalizability and reproducibility of our findings. Therefore, the development of new imaging modalities capable of providing absolute measurements of retinal blood flow will be essential for advancing research in this field and for potential clinical application. Third, although the moderate NPDR group was relatively small, the trend test was chosen because of its robustness to unequal group sizes and its suitability for ordered severity analyses. Finally, our interpretation emphasizes the dissociation between arterial hyperperfusion and ischemia in the capillaries; however, the present study did not include direct assessment of capillary nonperfusion using FA or OCTA. Therefore, the association between retinal arterial blood flow and the extent or distribution of capillary dropout could not be directly evaluated, and future studies that combine LDV with OCTA are required to validate this hypothesis. In particular, simultaneous assessment of deep capillary plexus nonperfusion may help clarify the dissociation between arterial hyperperfusion and capillary ischemia suggested by our findings.

In conclusion, to the best of our knowledge, retinal arterial blood flow increases with disease severity in T2D-related DR, suggesting that measuring blood flow in the retinal arterioles may provide useful insights into hemodynamic alterations associated with disease progression.

## Supplementary Information


Supplementary Information.


## Data Availability

The datasets used and/or analyzed during the current study are available from the corresponding author on reasonable request.
